# Bet v 1 and homologous food allergens are similarly processed by antigen-presenting cells but differ in T cell reactivity

**DOI:** 10.1186/2045-7022-4-S2-O12

**Published:** 2014-03-17

**Authors:** Claudia Kitzmueller, Nora Zulehner, Anargyros Roulias, Peter Briza, Fatima Ferreira, Barbara Bohle

**Affiliations:** 1 Medical University Vienna, Pathophysiology and Allergy Research, Vienna, Austria; 2 University of Salzburg, Molecular Biology, Salzburg, Austria

## Background

Various plant foods, e.g. apple and celery, express proteins that are homologues of the major birch-pollen allergen Bet v 1, e.g Mal d 1 and Api g 1. The proteins have 63% and 72% sequence similarity with Bet v 1 and share with it a common 3-dimensional structure. Despite this great molecular similarity, Bet v 1 is the only one among its homologues with the ability to sensitise atopic individuals. The aim of this study was to assess whether differences in the uptake and processing by antigen-presenting cells and in the presentation to T cells could be responsible for Bet v 1's ability to sensitise.

## Methods

Uptake of allergens by PBMC, surface binding to and degradation by monocyte-derived dendritic cells (mdDC) were assessed. Peptides derived from digestion of Bet v 1, Mal d 1 and Api g 1 by endo-lysosomal extracts were analysed by mass spectrometry. Epitope-specificity of allergen-specific T cell lines from birch pollen-allergic individuals with associated food-allergies was mapped using synthetic 12-mer peptides. Binding of allergen-derived peptides by HLA class II molecules was analysed *in silico*.

## Results

Significant differences were found neither in surface binding, in the kinetics of uptake by PBMC, the intracellular degradation by mdDC nor in the degradation by endo-lysosomal extracts. An immunodominant T cell epitope was found only in Bet v 1, but could not be referred to preferential binding to the most common HLA class II molecules.

## Conclusion

The ability of Bet v 1 to sensitise is not conferred by differential antigen-processing but might stem from differences in T cell reactivity and the diverse routes of uptake of the aeroallergen Bet v 1 and the food allergens.

